# Availability of, Barriers to Performing, and Educational Practices of Interventional Procedures for Refractory Pain in Cancer Patients: A Nationwide Survey of Designated Cancer Hospitals in Japan

**DOI:** 10.1089/pmr.2024.0028

**Published:** 2024-12-09

**Authors:** Yoshihisa Matsumoto, Yuko Uehara, Akio Mizushima, Toshifumi Kosugi, Miyuki Sone, Naoki Nakamura, Mitsunori Miyashita, Tatsuya Morita, Takuhiro Yamaguchi, Eriko Satomi

**Affiliations:** ^1^ Department of Palliative Therapy, Cancer Institute Hospital of Japanese Foundation for Cancer Research, Tokyo, Japan.; ^2^ Department of Palliative Medicine, National Cancer Center Hospital East, Kashiwa, Japan.; ^3^ Department of Palliative Medicine, Juntendo University Graduate School of Medicine, Tokyo, Japan.; ^4^ Department of Palliative Care, Saga-ken Medical Centre Koseikan, Saga, Japan.; ^5^ Department of Diagnostic Radiology/Interventional Radiology Center, National Cancer Center Hospital, Tokyo, Japan.; ^6^ Department of Radiation Oncology, St. Marianna University School of Medicine, Kawasaki, Japan.; ^7^ Department of Palliative Nursing, Health Sciences, Tohoku University Graduate School of Medicine, Sendai, Japan.; ^8^ Palliative and Supportive Care Division, Seirei Mikatahara General Hospital, Hamamatsu, Japan.; ^9^ Division of Biostatistics, Tohoku University School of Medicine, Sendai, Japan.; ^10^ Department of Palliative Medicine, National Cancer Center Hospital, Tokyo, Japan.

**Keywords:** refractory cancer pain, interventional procedures, availability, barriers, designated cancer hospital

## Abstract

**Background:**

Because of the limitations of pharmacological therapy, nonpharmacological therapies including intervention procedures are also important for quality of cancer pain management.

**Objective:**

To clarify the availability of, number performed, barriers to performing, and educational practices of four interventional procedures (celiac plexus neurolysis/splanchnic nerve neurolysis, phenol saddle block, epidural analgesia, and intrathecal analgesia) in designated cancer hospitals.

**Design:**

Cross-sectional survey.

**Setting:**

Designated cancer hospitals certified by the Japanese Government.

**Methods:**

We administered self-administered questionnaires to collect general information about the facility and interventional procedures for refractory cancer pain between January and April 2021.

**Results:**

Questionnaires were sent to 402 facilities, and we received 199 valid responses (49.5%). Regarding availability, 36.7%–59.8% of the designated cancer hospitals reported that each procedure was available. Regarding the frequency of these procedures performed in the past 3 years, medians ranged from 1 to 4 times for each procedure. Among designated cancer hospitals, 44.7–65.8% reported the presence of barriers. Barriers such as “no/few physicians technically able to perform the procedure,” “inability to follow-up after the procedure is implemented,” and “the facilities to which patients may be referred after implementation are limited” were particularly pronounced. Training and treatment practice were provided by 30.7–55.8% of designated cancer hospitals for the procedures. Moreover, 12.6%–15.6% of designated cancer hospitals educated physicians and nurses responsible for cancer care in the region about pain treatment for the procedures.

**Conclusions:**

Our findings suggest that designated cancer hospitals need to improve the availability, training, and education of interventional procedures.

## Introduction 

Pain is a common symptom associated with cancer that decreases the quality of life of patients.^1,2^ Pharmacological management is the basis of cancer pain treatment and may adequately relieve cancer pain.^3,4^ However, a recent meta-analysis revealed that the percentage of patients with pain remains high.^5^ Specifically, they reported that 66.4% of patients with advanced terminal cancer had pain and 38% of those with cancer of any stage had moderate-to-severe pain.

Pharmacological management of cancer pain in some patients remains insufficient. Refractory cancer pain, which is defined as pain that does not respond to standard pharmacological treatments,^6^ affects some patients. Individualized pharmacotherapy that considers the timing of treatment, individual characteristics, and nonpharmacological therapies is important for cancer-related pain. Among nonpharmacological therapies, the World Health Organization guidelines^7^ strongly recommend radiotherapy. Furthermore, authoritative guidelines^8–10^ include nonpharmacological therapies such as neural blockade, neuraxial infusion, electric neuromodulation, and cordotomy. Thus, an individualized multimodal approach is important in cancer pain management.^11,12^

The degree to which interventional procedures for patients with cancer pain are available and utilized remains unclear. Some nonpharmacological therapies, including neural blockade and neuraxial infusion, are effective for cancer pain. Previous studies reported that they are used to treat 3.8%–8% of patients with cancer.^13–15^ However, because there are several barriers to the implementation of these therapies,^16–21^ their availability is limited.

Information on the status, availability, and factors associated with the use of neural blockades and neuraxial infusions for cancer pain management is currently limited worldwide.^13–22^ Previous questionnaire surveys targeted palliative care physicians, referring physicians, and representatives of facilities at which such treatment was provided.^16–20,23^ However, a national survey of the individual professionals who completed these surveys has not yet been performed. In Japan, we previously performed a national survey of medical specialists to assess factors related to interventional procedures for refractory pain in patients with cancer.^24^ We found that implementation was low, and the relevant factors were the number of patients with cancer and pain seen annually, difficulty in gaining experience and acquiring skills due to the limited number of cases, not being permitted to implement strategies in their own facilities, and the difficulty of treating patients requiring the procedure because of a lack of time. However, the actual status of implementation at designated cancer hospitals, which should play a central role in the provision of cancer treatment, is unknown and requires further investigation.

The purpose of the present study was to clarify the availability of treatment, the number of cases performed, barriers to performing, and educational practices related to implementation of the following procedures using a nationwide survey of facilities at designated cancer hospitals: celiac plexus neurolysis/splanchnic nerve neurolysis (CPN), subarachnoid neurolytic block for perineal pain (phenol saddle block), epidural infusions of local anesthetic combined with opioids (Epi), and intrathecal analgesia (IA) for refractory cancer pain.

## Methods

### Study design

This cross-sectional study of designated cancer hospitals certified by the government was conducted in Japan. This survey was part of the “Research on the Construction of Systematic Pain Relief Methods in the Final Stage of Cancer Patients’ Medical Care” program. The study was approved by the Institutional Review Board of the National Cancer Center, Japan (6000-021). Formal approval of the study protocol by an Ethics Committee was not required according to Japanese national policies. All procedures were performed in accordance with the Declaration of Helsinki. We enclosed a letter explaining the purpose of the survey and explained that responses were voluntary. If the survey was filled out and returned, it was considered as consent.

### Participants and procedures

Between January and April 2021, a questionnaire on interventional procedures for refractory cancer pain was sent to all 402 designated cancer hospitals accredited by the Japanese Government as of December 2020. A letter of purpose, questionnaire, and self-addressed envelope were enclosed and mailed. A request was made in the letter of purpose for the recipient to reply within one month of receipt of the questionnaire. The questionnaires were designed so that they could be divided into departments within a facility for each procedure if necessary. Only one questionnaire per procedure was enclosed. A physician performing each procedure or a physician who was familiar with the procedure in the absence of a physician performing the procedure was asked to answer the questionnaire. The person in charge of each facility (Hospital Director or alternate) collected the questionnaires and returned them together. A postcard reminder was sent if the questionnaire was not returned within this timeframe.

### Measurements

In the present study, refractory pain was defined as pain for which patients, family members, or nurses requested further alleviation despite receiving appropriate pharmacological therapy. The self-administered questionnaires asked about general information about the facility, the number of specialists, and interventional procedures for refractory cancer pain. All participants were asked about the following background factors: type of hospital, number of beds, number of new patients with cancer per year, number of patients with cancer who died within the hospital per year, number of new patients treated by the palliative care team per year, existence of a palliative care unit, and the number of staff in each specialty. The self-administered questionnaires gathered data regarding the following interventional procedures for refractory cancer pain: CPN, phenol saddle block, Epi, and IA. The four procedures were selected because they have been investigated in previous studies,^16,18,20,23,24^ and were considered typical procedures for cancer pain in Japan. We did not distinguish between celiac plexus neurolysis and splanchnic nerve neurolysis from the viewpoint of performing neural blockades for upper abdominal pain, even though the techniques and injection sites of the neurolytic agents differ. Facilities reported whether they currently performed these four therapies by a multiple choice that consisted of three responses (“currently implementing in their own facilities,” “referring to other facilities for implementation,” and “not available”). Moreover, we assessed the number of procedures they had performed or referred in the past three years and the departments where the procedures were performed. Facilities also reported the presence of barriers related to the implementation of the four procedures (yes/no) and the degree of hindrance of each barrier item. The facilities also responded to whether their staff underwent education for each procedure (Yes/No).

The questions were based on previous studies^16–21,23^ and were developed through discussions among members of an expert group. Responses to the degree of hindrance of each barrier item were recorded on a four-point Likert scale with the following available responses: “not at all,” “a little,” “moderately,” and “greatly.”

### Analysis

Analyses were performed on the valid responses using descriptive statistics. We calculated the frequency of the responses for each item. In addition, we calculated 95% confidence intervals, where appropriate. We analyzed data where the percentage of missing data did not exceed 25%. All analyses were performed using SPSS version 25 (SPSS Inc., Chicago, IL, USA) and R version 4.3.3.

## Results

### Response rate

Questionnaires were sent to all 402 designated cancer hospitals. Valid responses were obtained from 199/402 facilities (49.5%).

### Characteristics

Participant characteristics are shown in Table 1. Of the 402 cancer-designed hospitals, 25.1% were university hospitals and 63.3% were designated hospitals for postgraduate clinical training. More than half of the hospitals had more than 600 beds and approximately one-third had palliative care units. The median number of patients with patients seen annually was 1895. The median number of patients with cancer who died in the hospital annually was 196. The median numbers of the four types of certified specialists working in the designated cancer hospital were: one palliative care physician, one pain clinician, six anesthesiologists, and one intervention radiologist.

**Table 1.** Participant Characteristics (*n* = 199)

Types of hospitals, *N* (%)		
University hospital	Yes	50 (25.1%)
Designated hospital for postgraduate clinical training	Yes	126 (63.3%)
Number of beds, N (%)		
<200		2 (1.0%)
200–399		23 (11.6%)
400–599		67 (33.7%)
600–799		65 (32.7%)
800–999		26 (13.1%)
1000+		14 (7.0%)
Missing		2 (1.0%)
Presence of a palliative care unit, N (%)	Yes	66 (33.2%)
Number of patients, median (IQR)		
Patients with cancer seen annually		1895 (1353, 2861.25)
Patients with cancer who died annually		196 (142, 298)
Certified specialists, median (IQR)		
Palliative care physician		1 (0, 2)
Pain clinician		1 (0, 2)
Anesthesiologist		6 (4, 11)
Intervention radiologist		1 (0, 3)

IVR, interventional radiology; SD, standard deviation; IQR, interquartile range.

### Implementation of interventional procedures

Table 2 shows the implementation of interventional procedures in designated cancer hospitals. The percentages of designated cancer hospitals that indicated “Currently implementing at own facility,” “Referring to other facilities for implementation,” and “Not available” for the various interventional procedures were as follows: CPN, 49.7%, 10.1%, and 38.2%; phenol saddle block, 43.7%, 10.1%, and 43.7%; Epi, 54.8%, 3.5%, and 39.2%; and IA, 30.7%, 6.0%, and 60.8%, respectively. Table 3 shows the number of implementations in the past three years. The median number of implementations (interquartile ranges) were: CPN, 4 (2–9); phenol saddle block, 2 (0–4); Epi, 3 (1–6); and IA, 1 (0–3). The numbers of designated cancer hospitals where 10 or more procedures were being performed were CPN, 22 (22.2%); phenol saddle block, five (5.7%); Epi, 19 (8.3%); and IA, four (6.6%), respectively.

**Table 2.** Implementation of Interventional Procedures at Designated Cancer Hospitals

	Celiac plexus neurolysis/splanchnic nerve neurolysis			Subarachnoid neurolytic block for perineal pain (phenol saddle block)			Epidural infusions of local anesthetic combined with opioids			Intrathecal analgesia		
	N	%	95% CI	N	%	95% CI	N	%	95% CI	N	%	95% CI
Currently implementing at own facility	99	49.7	42.6–56.9	87	43.7	36.7–50.9	109	54.8	47.6–61.8	61	30.7	24.3–37.6
Referring to other facilities for implementation	20	10.1	6.2–15.1	20	10.1	6.2–15.1	7	3.5	1.4–7.1	12	6.0	3.2–10.3
Not available	76	38.2	31.4–45.3	87	43.7	36.7–50.9	78	39.2	32.4–46.3	121	60.8	53.7–67.6
Missing	4	2.0		5	2.5		5	2.5		5	2.5	

CI, confidence interval.

**Table 3.** Number of Implementations in the Past Three Years

	Celiac plexus neurolysis/splanchnic nerve neurolysis (*n* = 99)			Subarachnoid neurolytic block for perineal pain (phenol saddle block) (*n* = 87)			Epidural infusions of local anesthetic combined with opioids (*n* = 109)			Intrathecal analgesia (*n* = 61)		
	N	%	95% CI	N	%	95% CI	N	%	95% CI	N	%	95% CI
Median (IQR)	4 (2–9)			2 (0–4)			3 (1–6)			1 (0–3)		
0	9	9.1	4.2–16.6	23	26.4	17.6–37.0	13	11.9	6.5–19.5	19	31.1	20.0–44.3
1–4	40	40.4	30.7–50.7	45	51.7	40.8–62.6	57	52.3	42.5–61.9	29	47.5	34.6–60.7
5–9	26	26.3	17.9–36.1	12	13.8	7.3–22.9	18	16.5	10.1–24.8	7	11.5	4.7–22.2
10–19	15	15.2	8.7–23.8	3	3.4	0.7–9.7	13	11.9	6.5–19.5	3	4.9	1.0–13.7
20–29	3	3.0	0.6–8.6	2	2.3	0.3–8.1	0	0	0.0–3.3	1	1.6	0.0–8.8
30+	4	4.0	1.1–8.6	0	0	0.0–4.2	6	5.5	2.0–11.6	0	0	0.0–5.9
Missing	2	2.0		2	2.3		2	1.8		2	3.3	

All procedures were most commonly performed at the Anesthesiology/Pain Clinic (CPN, 67.7%; phenol saddle block, 81.6%; Epi, 86.2%; and IA, 72.1%), followed by the Department of Palliative Medicine. CPN was also performed by the Department of Radiology/IVR/Radiation Therapy (12.1%) and the Department of Internal Medicine (13.1%).

### Barriers to the implementation of procedures

**FIG. 1.**
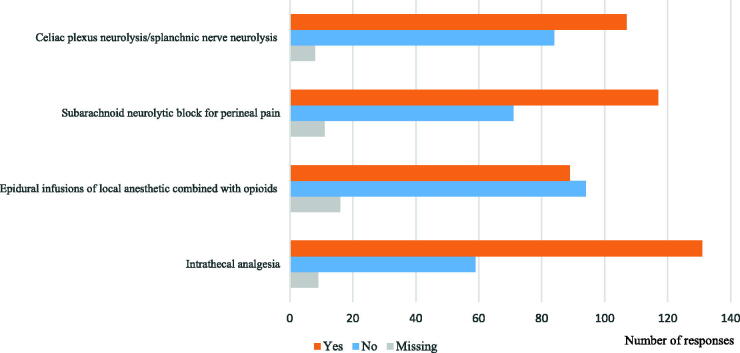
Presence of barriers to performing each procedure.

Figure 1 shows the barriers to performing each procedure. The percentage of designated cancer hospitals that indicated that barriers to performing the procedure were present were: CPN, 53.8%; phenol saddle block, 58.8%; Epi, 44.7%; and IA, 65.8%.

Table 4 shows barriers to the implementation of procedures. The items for which more than half of the designated cancer hospitals reported moderate or greater barriers were as follows: “No/few physicians technically able to perform the procedures” for CPN, phenol saddle block, and IA; “Not permitting physicians technically capable of performing the procedure to perform it due to their working situation” for CPN, phenol saddle block, Epi, and IA; “Inability to follow up after the procedure is implemented” for IA; and “The facilities to which patients may be referred after implementation are limited” for Epi and IA.

**Table 4.** Barriers to the Implementation of the Procedures at Designated Cancer Hospitals

	Celiac plexus neurolysis/splanchnic nerve neurolysis (*n* = 107)					Subarachnoid neurolytic block for perineal pain (*n* = 117)					Epidural infusions of local anesthetic combined with opioids (*n* = 89)					Intrathecal analgesia (*n* = 131)				
Barriers	Not at all	A little	Moderately	Greatly	Missing	Not at all	A little	Moderately	Greatly	Missing	Not at all	A little	Moderately	Greatly	Missing	Not at all	A little	Moderately	Greatly	Missing
Lack of a health professional who can determine indications for the procedure N (%)	29 (27.1)	38 (35.5)	17 (15.9)	20 (18.7)	3 (2.8)	38 (32.5)	38 (32.5)	18 (15.4)	19 (16.2)	4 (3.4)	28 (31.5)	33 (37.1)	19 (21.3)	7 (7.9)	2 (2.2)	40 (30.5)	34 (26.0)	35 (26.7)	16 (12.2)	6 (4.6)
No/few physicians technically able to perform the procedure N (%)	13 (12.1)	17 (15.9)	21 (19.6)	55 (51.4)	1 (0.9)	21 (17.9)	27 (23.1)	24 (20.5)	44 (37.6)	1 (0.9)	32 (36.0)	18 (20.2)	20 (22.5)	18 (20.2)	1 (1.1)	21 (16.0)	32 (24.4)	34 (26.0)	42 (32.1)	2 (1.5)
Not permitting physicians technically capable of performing it to perform the procedure due to their working situation N (%)	21 (19.6)	21 (19.6)	20 (18.7)	39 (36.4)	6 (5.6)	28 (23.9)	21 (17.9)	26 (22.2)	35 (29.9)	7 (6.0)	17 (19.1)	17 (19.1)	21 (23.6)	33 (37.1)	1 (1.1)	28 (21.4)	25 (19.1)	34 (26.0)	37 (28.2)	7 (5.3)
Inability to follow up after the procedure is implemented N (%)	29 (27.1)	33 (30.8)	20 (18.7)	22 (20.6)	3 (2.8)	30 (25.6)	33 (28.2)	32 (27.4)	18 (15.4)	4 (3.4)	22 (24.7)	25 (28.1)	22 (24.7)	18 (20.2)	2 (2.2)	24 (18.3)	34 (26.0)	32 (24.4)	37 (28.2)	4 (3.1)
Lack of/unavailability of equipment, facilities, or chemicals to perform the procedure N (%)	38 (35.5)	29 (27.1)	10 (9.3)	26 (24.3)	4 (3.7)	32 (27.4)	28 (23.9)	22 (18.8)	33 (28.2)	2 (1.7)	51 (57.3)	24 (27.0)	5 (5.6)	7 (7.9)	2 (2.2)	47 (35.9)	30 (22.9)	20 (15.3)	30 (22.9)	4 (3.1)
No facilities where the procedure is performed in a region that can be referred from own facility N (%)	39 (36.4)	30 (28.0)	20 (18.7)	15 (14.0)	3 (2.8)	49 (41.9)	27 (23.1)	26 (22.2)	11 (9.4)	4 (3.4)	38 (42.7)	27 (30.3)	15 (16.9)	7 (7.9)	2 (2.2)	41 (31.3)	35 (26.7)	28 (21.4)	22 (16.8)	5 (3.8)
Lack of information on facilities where the procedure is performed in the region that patients can be referred from their own facility N (%)	39 (36.4)	30 (28.0)	18 (16.8)	17 (15.9)	3 (2.8)	45 (38.5)	27 (23.1)	24 (20.5)	19 (16.2)	2 (1.7)	34 (38.2)	29 (32.6)	14 (15.7)	11 (12.4)	1 (1.1)	45 (34.4)	29 (22.1)	29 (22.1)	23 (17.6)	5 (3.8)
No referrals of eligible patients from other facilities or your own facility N (%)	25 (23.4)	40 (37.4)	21 (19.6)	17 (15.9)	4 (3.7)	31 (26.5)	43 (36.8)	23 (19.7)	16 (13.7)	4 (3.4)	23 (25.8)	33 (37.1)	22 (24.7)	9 (10.1)	2 (2.2)	30 (22.9)	46 (35.1)	30 (22.9)	21 (16.0)	4 (3.1)
Inability to respond when complications arise from the procedure N (%)	30 (28.0)	36 (33.6)	22 (20.6)	16 (15.0)	3 (2.8)	36 (30.8)	33 (28.2)	24 (20.5)	20 (17.1)	4 (3.4)	35 (39.3)	24 (27.0)	14 (15.7)	14 (15.7)	2 (2.2)	29 (22.1)	42 (32.1)	26 (19.8)	30 (22.9)	4 (3.1)
Not permitted to perform the procedure at the facility N (%)	62 (57.9)	22 (20.6)	8 (7.5)	12 (11.2)	3 (2.8)	59 (50.4)	26 (22.2)	16 (13.7)	13 (11.1)	3 (2.6)	56 (62.9)	17 (19.1)	7 (7.9)	7 (7.9)	2 (2.2)	63 (48.1)	35 (26.7)	17 (13.0)	12 (9.2)	4 (3.1)
Lack of cooperation from relevant medical departments N (%)	41 (38.3)	32 (29.9)	17 (15.9)	14 (13.1)	3 (2.8)	46 (39.3)	28 (23.9)	21 (17.9)	18 (15.4)	4 (3.4)	30 (33.7)	25 (28.1)	18 (20.2)	14 (15.7)	2 (2.2)	52 (39.7)	36 (27.5)	18 (13.7)	20 (15.3)	5 (3.8)
Unprofitability of the procedure N (%)	50 (46.7)	36 (33.6)	9 (8.4)	9 (8.4)	3 (2.8)	56 (47.9)	32 (27.4)	14 (12.0)	10 (8.5)	5 (4.3)	38 (42.7)	26 (29.2)	11 (12.4)	12 (13.5)	2 (2.2)	52 (39.7)	47 (35.9)	14 (10.7)	13 (9.9)	5 (3.8)
The facilities to which patients may be referred after implementation are limited N (%)	—	—	—	—	—	—	—	—	—	—	22 (24.7)	19 (21.3)	25 (28.1)	21 (23.6)	2 (2.2)	29 (22.1)	31 (23.7)	34 (26.0)	32 (24.4)	5 (3.8)

### Education for each treatment for pain

Table 5 shows information regarding education about each treatment for pain. The percentages of designated cancer hospitals that responded that they provided training and practice for the following treatments at their own facility were: CPN, 41.2%; phenol saddle block, 37.3%; Epi, 55.8%; and IA, 30.7%. The percentages of designated cancer hospitals that responded that they provided training for determining indications for the following treatments at their own facility (regardless of practice of the procedure) were: CPN, 53.8%; phenol saddle block, 50.8%; Epi, 58.8%; and IA, 41.7%. The percentages of designated cancer hospitals that responded that they educated physicians and nurses in charge of cancer care in their own facility about the following treatments were: CPN, 28.1%; phenol saddle block, 21.6%; Epi, 27.1%; and IA, 20.6%. The percentages of designated cancer hospitals that responded that they educated physicians and nurses in charge of cancer care in the region about the following treatments for pain were: CPN, 15.6%; phenol saddle block, 12.6%; Epi, 14.6%; and IA, 13.1%.

**Table 5.** Education of Each Pain Treatment

	Celiac plexus neurolysis/splanchnic nerve neurolysis			Subarachnoid neurolytic block for perineal pain			Epidural infusions of local anesthetic combined with opioids			Intrathecal analgesia		
	Yes	No	Missing	Yes	No	Missing	Yes	No	Missing	Yes	No	Missing
Providing training and treatment practice at their own facility N (%)	82 (41.2)	86 (43.2)	31 (15.6)	75 (37.7)	87 (43.7)	37 (18.6)	111 (55,8)	55 (27.6)	33 (16.6)	61 (30.7)	93 (46.7)	45 (22.6)
Providing training for determining indications for the treatment at their own facility (regardless of practice of the procedure) N (%)	107 (53.8)	62 (31.2)	30 (15.1)	101 (50.8)	61 (30.7)	37 (18.6)	117 (58.8)	49 (24.6)	33 (16.6)	83 (41.7)	71 (35.7)	45 (22.6)
Educating physicians and nurses in charge of cancer care in their own facility about the treatment N (%)	56 (28.1)	113 (56.8)	30 (15.1)	43 (21.6)	119 (59.8)	37 (18.6)	54 (27.1)	112 (56.3)	33 (16.6)	41 (20.6)	113 (56.8)	45 (22.6)
Educating physicians and nurses in charge of cancer care in the region about the treatment N (%)	31 (15.6）	138 (69.3)	30 (15.1)	25 (12.6)	137 (68.8)	37 (18.6)	29 (14.6)	137 (68.8)	33 (16.6)	26 (13.1)	128 (64.3)	45 (22.6)

## Discussion

In this study, we conducted a nationwide survey of designated cancer hospitals. Our findings clarified the availability, status of implementation, barriers, and status of education regarding the implementation of interventional procedures for refractory pain in patients with cancer.

The percentage of designated cancer hospitals that responded that they were able to perform the procedures was low (30.7%–54.8%), and including hospitals that responded positively to “Referring to other facilities for implementation,” the percentages of hospitals that were available for the procedures were not high enough (36.7%–59.8%). Even for the facilities that responded that they were able to perform the procedures. In the past three years, more than half of the facilities had fewer than five cases for all procedures except CPN (49.5%) and the percentage of facilities with 0 cases for the procedures were 9.1%–31.1%. Previous surveys of specialist pain services examined the availability of interventional procedures. In the UK, CPN, intrathecal neurolysis, spinal analgesia procedures, EPI, IA, and both EPI and IA were available at 24.5%, 24.5%, 85.8%, 22%, 18%, and 45% of facilities, respectively.^16^ In Japan, CPN, intrathecal neurolysis, EPI, and IA procedures were available at 66%, 67.4%, 88.2%, and 54.2% of facilities.^23^ Thus, despite a certain number of pain specialist facilities being capable of providing interventional analgesia for patients with cancer, pain clinicians have limited opportunities to perform these procedures.^21,24^ In the present study of accredited facilities that collectively provided care to patients with cancer, the availability rate was found to be low. We previously reported that the number of procedures performed by specialists may be insufficient from a patient perspective.^24^ The present study supports our previous study.

Factors related to the implementation of interventional procedures warrant further study. Previous studies reported the following barriers to the implementation of specialist pain management, such as neural blockade and neuraxial infusion: underutilization of specialists;^16,17^ access issues/geographical issues;^18,19^ inter-facility issues;^19^ inability to get appointments;^20^ need for repeating procedures;^20^ cost issues;^17,18,21^ short survival of patients following referral to palliative care services;^21^ time (on the part of the specialist) for evaluation and discussion;^16,21^ complexity;^21^ continuity issues, such as the handling of pumps and catheters, creating a pump, procurement of drugs, and management at home;^21^ inexperience of palliative care physicians;^18^ perception issues among palliative care physicians (interest or lack of awareness of potential benefits);^18,21^ and lack of training for specialists.^21^ In our survey of medical specialists, we also found that the number of patients with cancer and pain seen annually, difficulty in gaining experience, lack of time, and lack of institutional acceptance were associated with low implementation of procedures.^24^ The first three factors are consistent with previously reported associated factors: involvement of specialists in palliative care,^16^ time on the part of the specialist for evaluation and discussion,^16^ and lack of training for specialists.^21^ In the present study, with the exception of Epi, more than half of the facilities had some barrier to performing the included procedures. Specifically, “No/few physicians technically able to perform the procedure,” “Not permitting physicians technically capable of performing the procedure to perform it due to their working situation,” “Inability to follow up after the procedure is implemented,” and “The facilities to which patients may be referred after implementation are limited” were often recognized as barriers.

Our findings suggest that training and allocation of time for specialists, along with collaboration with regional facilities, are crucial for the widespread adoption of the procedures. However, our findings suggest that many designated cancer hospitals are unable to offer sufficient education for medical staffs in their own facility and specialists, and dissemination efforts to regional facilities and medical staff are also insufficient.

Our findings suggest the following measures to increase the number of interventional procedures performed. First, specialists performing the procedures need to increase their experience in treating such patients. To increase their experience, several strategies may be effective, including further specialization for the treatment of cancer pain, a region-wide networking system for identifying potential candidates for interventional procedures, and establishing designated teaching facilities. Second, the effective use of time by specialists to perform procedures in palliative medicine may increase their implementation. Increasing the time spent in palliative medicine may compensate for lack of experience. In a 2007 survey conducted among lead anesthetists in UK pain clinics,^16^ it was found that joint consulting arrangements were rare, and only 25% of anesthetists’ job plans allocated time specifically for palliative medicine referrals. However, there was a notable positive correlation between the number of referrals and these allocations. Thus, promoting opportunities for specialists to be involved in palliative medicine may in turn increase the number of interventional procedures performed. Third, efforts are needed to educate palliative care physicians to serve as bridges to connect patients to specialists. A previous survey of home hospice physicians and oncologists revealed that they had knowledge of the implementation of procedures;^24^ however, they lacked experience or were unable to refer patients to specialists. Previous studies have also reported a lack of experience and awareness among palliative care physicians.^18,21^ Further education and awareness of the indications for and effects of interventional therapies among palliative care physicians are needed. Moreover, physicians and nurses in charge of cancer care in the facilities and in the region should be educated about pain treatment.

Given the scarcity of evidence-based interventional procedures, specialists may encounter challenges in justifying the procedure. Furthermore, palliative care physicians, who often serve as intermediaries, may struggle to propose procedures confidently and obtain institutional approval. Further research is warranted to assess the efficacy of these interventional procedures.

### Limitations

The present study has several limitations. First, the valid response rate for the designated cancer hospitals was 49.5%, which may not reflect the overall situation. However, the response rate was sufficient for surveys in individual hospitals. Second, Japan has a universal health insurance system, no restrictions on access to medical facilities, and relatively small geographical size; thus, our findings may not be applicable to other countries. Moreover, we did not inquire about geographical barriers, which have been identified as barriers in previous studies. Finally, we did not investigate electric neuromodulation, cordotomy, and radiofrequency ablation.^25,26^ However, electric neuromodulation and cordotomy were not yet commonly performed for cancer pain in Japan, and we believe that radiofrequency ablation could be performed at the facilities that can perform the four procedures in this survey.

## Conclusion

In the present study, we found that designated cancer hospitals were not fully available for interventional procedures and had inadequate provision of training and education for interventional procedures. It is important to take measures to ensure that interventional procedures for refractory cancer pain are sufficiently utilized in designated cancer hospitals such as increasing specialist’s experience in procedure, increasing the effective use of time by specialists and educating palliative care physicians to serve as bridges.

## Ethics Approval and Consent to Participate

The study was approved by the Institutional Review Board of the National Cancer Center, Japan (6000-021). Formal approval of the study protocol by an Ethics Committee was not required according to the Japanese national policies. All procedures were performed in accordance with the Declaration of Helsinki. We enclosed a letter explaining the purpose of the survey and explained that responses were voluntary. If the survey was filled out and returned, it was considered as consent.

## Abbreviations Used

CI: Confidence interval
CPN: Celiac plexus neurolysis/splanchnic nerve neurolysis
Epi: Epidural infusions of local anesthetic combined with opioids
IA: Intrathecal analgesia
IQR: Interquartile range
IVR: Interventional radiology
phenol saddle block: Subarachnoid neurolytic block for perineal pain
SD: Standard deviation
